# Characterization and expression analysis of *Galnts* in developing *Strongylocentrotus purpuratus* embryos

**DOI:** 10.1371/journal.pone.0176479

**Published:** 2017-04-27

**Authors:** Amber L. Famiglietti, Zheng Wei, Thomas M. Beres, Adina L. Milac, Duy T. Tran, Divya Patel, Robert C. Angerer, Lynne M. Angerer, Lawrence A. Tabak

**Affiliations:** 1 Section on Biological Chemistry, National Institute of Dental and Craniofacial Research, National Institutes of Health, Bethesda, MD, United States of America; 2 Developmental Mechanisms Section, National Institute of Dental and Craniofacial Research, National Institutes of Health, Bethesda, MD, United States of America; 3 Department of Bioinformatics and Structural Biochemistry, Institute of Biochemistry of the Romanian Academy, Splaiul Independentei 296, Bucharest, Romania; Laboratoire Arago, FRANCE

## Abstract

Mucin-type O-glycosylation is a ubiquitous posttranslational modification in which N-Acetylgalactosamine (GalNAc) is added to the hydroxyl group of select serine or threonine residues of a protein by the family of UDP-GalNAc:Polypeptide N-Acetylgalactosaminyltransferases (GalNAc-Ts; EC 2.4.1.41). Previous studies demonstrate that O-glycosylation plays essential roles in protein function, cell-cell interactions, cell polarity and differentiation in developing mouse and *Drosophila* embryos. Although this type of protein modification is highly conserved among higher eukaryotes, little is known about this family of enzymes in echinoderms, basal deuterostome relatives of the chordates. To investigate the potential role of GalNAc-Ts in echinoderms, we have begun the characterization of this enzyme family in the purple sea urchin, *S*. *purpuratus*. We have fully or partially cloned a total of 13 genes (*SpGalnt*s) encoding putative sea urchin SpGalNAc-Ts, and have confirmed enzymatic activity of five recombinant proteins. Amino acid alignments revealed high sequence similarity among sea urchin and mammalian glycosyltransferases, suggesting the presence of putative orthologues. Structural models underscored these similarities and helped reconcile some of the substrate preferences observed. Temporal and spatial expression of *SpGalnt* transcripts, was studied by whole-mount *in situ* hybridization. We found that many of these genes are transcribed early in developing embryos, often with restricted expression to the endomesodermal region. Multicolor fluorescent *in situ* hybridization (FISH) demonstrated that transcripts encoding *SpGalnt7-2* co-localized with both *Endo16* (a gene expressed in the endoderm), and *Gcm (*a gene expressed in secondary mesenchyme cells) at the early blastula stage, 20 hours post fertilization (hpf). At late blastula stage (28 hpf), *SpGalnt7-2* message co-expresses with *Gcm*, suggesting that it may play a role in secondary mesenchyme development. We also discovered that morpholino-mediated knockdown of *SpGalnt13* transcripts, results in a deficiency of embryonic skeleton and neurons, suggesting that mucin-type O-glycans play essential roles during embryonic development in *S*. *purpuratus*.

## Introduction

Mucin-type O-glycosylation is an essential protein modification in which N-Acetygalactosamine (GalNAc) is added to the hydroxyl group of select serine or threonine residues of proteins by a family of enzymes termed UDP-GalNAc:Polypeptide N-Acetylgalactosaminyltransferases (GalNAc-Ts; EC 2.4.1.41) [1]. A total of 20 members of the GalNAc-T family have been identified in humans, 19 members in mice [1] and 12 members in *Drosophila* [2]. All GalNAc-Ts are type II transmembrane proteins, consisting of an N-terminal cytoplasmic tail, a hydrophobic region, a conserved catalytic domain and, with one exception, a lectin domain. These enzymes exhibit complex *in vitro* preferences for substrates, exemplified by a subset of GalNAc-Ts that prefer to add GalNAc to unmodified substrates (“peptide transferases”) and others that display preferences for substrates in which GalNAc has been previously added (“glycopeptide preferring transferases”) [3, 4].

Mucin-type O-glycosylation has been shown to play diverse roles in development and normal physiologic processes. For example, cell surface mucin-type O-glycans influence a number of cellular properties including cell-cell interactions, cell differentiation, cell adhesion, and cell polarity [2]. The importance of *Galnt1* in the development of heart valves in mice has recently been described. Loss of *Galnt1* was shown to reduce levels of ADAMTS1 and ADAMTS5, alter extracellular matrix processing and increase BMP/MAPK signaling, resulting in increased cell proliferation and improper regulation of valvulogenesis [5]. Furthermore, adult mice lacking this GalNAc-T family member exhibited valvular stenosis and cardiac impairment [5]. *Galnt1* was also found to regulate proper development of mouse salivary glands by regulating the secretion of the basement membrane [6]. Mice deficient in the galactosyltransferase T-synthase fail to build core 1 O-glycans (Galactose β1,3 GalNAc α1-O-Thr/Ser), which results in fatal embryonic hemorrhage [7]. A mutation in one member of the human GalNAc-T family (*GALNT3)* underlies the disease familial tumoral calcinosis [8].

Although this type of posttranslational modification is evolutionarily conserved throughout the animal kingdom, very little is known about this process in marine invertebrates [9]. Pioneering work by Lennarz and colleagues demonstrated the importance of N-linked glycosylation in early embryonic development in sea urchins. There is a marked increase in the rate of glycoprotein synthesis just prior to embryonic gastrulation [10–12]. Inhibition of N-linked glycosylation through the use of a drug, tunicamycin, resulted in arrested embryonic development at the early gastrula stage, suggesting that N-linked glycoproteins are essential for normal cell migration and gastrulation [13]. Sulphated, O-linked glycans that decorate the sea urchin 350 kDa egg receptor for sperm have also been shown by a variety of in vitro assays to be involved in the binding of acrosome-reacted sperm [14] (Reviewed in [15]).

In the current study, we have performed the first characterization of the GalNAc-T family in the purple sea urchin, *S*. *purpuratus*. We identified 13 genes encoding putative GalNAc-Ts through an *in silico* search of the sea urchin genome database and these genes were confirmed by PCR amplification using *S*. *purpuratus* cDNA. In vitro enzymatic activity assays demonstrated functional transferase activity for five isoforms (SpGalNAc-T1, SpGalNAc-T2, SpGalNAc-T7, SpGalNAc-T7-1, and SpGalNAc-T7-2) and computational structural models of these isoforms in complex with several substrate peptides revealed the structural basis of specific substrate preferences. Sequence analysis revealed high levels of similarity between sea urchin and mammalian GalNAc-T isoforms, confirming that mucin-type O-glycosylation is an evolutionarily conserved process among deuterostomes. We found that morpholino-mediated knockdown SpGalNAc-T13 resulted in skeletal and neuron deficiency, suggesting that mucin-type O-glycans are required for proper early development.

## Materials and methods

### 1. Retrieval of GalNAc-T protein sequences and cloning of Galnt genes in sea urchin

Amino acid sequences for human and mouse GalNAc-T proteins were extracted from the UniProt Database [16, 17]. Putative sea urchin *Galnt* genes were found either from annotated predictions of the sea urchin genome [18] or by a BLAST search of the sea urchin genome sequence with mammalian homologs as targets. Domain assignment was performed using the Conserved Domain Search service (CD-Search) [19] from NCBI Conserved Domain Database [20]. Sequence alignments were confirmed using Multalin [21] and Clustal Omega [22]. Each putative sea urchin *Galnt* sequence was isolated by PCR amplification of 24-hour, 36-hour and 48-hour sea urchin embryo cDNAs or an Invitrogen custom cDNA library (pCMV-SPORT6.1).

### 2. Evaluation of sea urchin GalNAc-T sequence variability

Variability analysis of the resulting sequence alignments for both catalytic and lectin domains were carried out using the PVS tool [23]. Sequence variability score was mapped on the structure of the first human x-ray structure of a GalNAcT-2 with PDB code 2FFU.pdb [24] and presented using the Beta Coloring scale in VMD [25–27] (http://www.ks.uiuc.edu/Research/vmd/).

### *3*. Phylogenetic analysis

To ascertain more accurately the similarity relationships between GalNAc-T isoforms and trace the evolution of different SpGalNAc-T isoforms, we performed a detailed phylogenetic analysis including 20 human, 19 mouse, 12 Drosophila and 13 sea urchin (64 sequences in total, alignment length 350 residues). This analysis focused only on the catalytic domain due to its crucial functional role and the extremely high sequence variability in the lectin domain, which makes sequence alignment uncertain in this region.

Phylogenetic analysis was performed using two likelihood-based methods of phylogenetic inference. The first is maximum-likelihood method as implemented in PhyML 3.0 (http://www.atgc-montpellier.fr/phyml/) [28], using the LG matrix aminoacids substitution model [29]. The starting tree was generated by BioNJ algorithm [30, 31] and tree topology search was performed using Nearest Neighbor Interchanges (NNIs) algorithm. To obtain statistical support, bootstrapping was applied for 1000 iterations.

The second method uses a Bayesian approach using MrBayes v3.1.2 [32] as implemented in Armadillo v1.1 (http://www.bioinfo.uqam.ca/armadillo), a novel workflow platform dedicated to designing and conducting phylogenetic studies [33]. One cold and two incrementally heated Markov chain Monte Carlo (MCMC) chains were run for 100,000 generations. Trees were sampled every 10 generations. MCMC runs were repeated twice to avoid spurious results. The first 2500 trees before stationarity were discarded as burn-in, and the remaining trees were used to construct the majority-rule consensus trees. The average standard deviation of split frequencies between the two runs was 0.0691.

Optimal trees obtained by both methods were visualised and adjusted for size and image resolution using MEGA7 software [34].

### 4. Homology modeling of SpGalNAc-T isoforms

For each of the SpGalNAc-T isoforms T1, T2, T7, T7-1 and T7-2 we built 3D models in extended (open) and compact (closed) states, in complex with EA2 and Muc5Ac-13 peptides (24 models in total). The X-ray structures used as templates were: 2FFU.pdb—enzyme in extended conformation and in complex with EA2 peptide [24] and 5AJP.pdb—enzyme in compact conformation and in complex with Muc5Ac-13 glycopeptide [35]. Structural models were built using Modeller v. 9.11 [36] and analyzed using Pymol (The PyMOL Molecular Graphics System, Version 1.8 Schrödinger, LLC.).

The structural superposition of the two templates indicates identical conformation of the peptide main chain along seven amino acids (three residues upstream and downstream of the glycosylation site), suggesting that the peptides have similar conformation in both open and closed states of the enzyme. Given the high sequence similarity between targets and templates (identity between 37 and 71%, similarity between 62 and 88% along ~500 aa alignment), we expect high accuracy of the resulting 3D models, i.e. comparable to the resolution of experimental structures used as templates (1.65Ǻ).

### 5. Generation of sea urchin GalNAc-T secretion constructs

The truncated coding region of each *SpGalnt* was amplified by PCR and cloned into a pIMKF4 expression vector. *SpGalnt1* cDNA spanning amino acid residues 68 to 597 and *SpGalnt2* cDNA spanning amino acid residues 37 to 565 were each cloned in frame using MluI and SacI sites. *SpGalnt7* cDNAs spanning amino acid residues 53 to 671, *SpGalnt7-1* cDNA spanning amino acid residues 31 to 607, and *SpGalnt13* cDNA spanning amino-acid residues 264- to 746 were each cloned in frame using MluI and AgeI sites. *SpGalnt7-2* cDNA spanning amino acid 38 to 605 was cloned in frame using MluI and NotI sites.

### 6. In vitro enzymatic activity assays of secreted recombinant SpGalNAc-Ts

Each expression vector was transfected into COS7 cells as previously described [37]. After 72 hours, cell culture media containing secreted protein (SpGalNAc-T1 and SpGalNAc-T2) were collected and purified using FLAG-affinity agarose (Sigma A2220). Un-secreted proteins (SpGalNAc-T7, SpGalNAc-T7-1, and SpGalNAc-T7-2) were collected from cell lysates. Approximately 24 hours post transfection, cell culture media were removed and cells were scraped off, washed with 1X PBS, resuspended in 1% Triton in 1X PBS plus 1% Halt Protease Inhibitor cocktail, and sonicated. Sonication supernatant was then collected and purified using FLAG-affinity agarose (Sigma A2220).

Functional expression assays were performed as previously described [37], with the exception that all reactions were carried out at 37°C for three hours. Triplicate reactions were used to test all putative enzymes against peptide and glycopeptide substrates: EA2 (PTTDSTTPAPTTK) derived from rat submandibular gland mucin[38], MUC5AC (GTTPSPVPTTSTTSAP) derived from human MUC5AC mucin [39], MUC5AC-3 (MUC5AC- glycosylated at T^3^) [40], MUC5AC-13 (MUC5AC-glycosylated at T^13^) [40], MUC5AC-3/-13 (MUC5AC-glycosylated at both T^3^ and T^13^) [40], SpDelta (PNLRATSSPITN FGLSDTMQL) and SpNotch (PVILTSPPETTLAVVPTTTESPRC) based on SPU_016128 and SPU_014131 sequences [41], respectively. Background values acquired from COS7 cells transfected with vector alone were subtracted from each experimental value. All negative experimental values were denoted as zero counts. Whisker and box plots of enzymatic activity (dpm/3hr) with background previously subtracted show the variability in activity levels observed among three independent transfections. Error bars indicate relative maxima and minima of all combined data points collected from assays performed in triplicate from three separate transfections.

### 7. Single and multicolor fluorescent mRNA in situ hybridizations

Whole-mount mRNA *in situ* hybridization (WMISH) was carried out as previously reported [42]. Three-color fluorescent *in situ* hybridization was performed as described previously [43]. Images shown are representative of three independent hybridization experiments. Multicolor WMISH contained a *SpGalnt7-2* probe labeled with digoxigenin and detected with Cy5-TSA, a *Gcm* probe labeled with dNP and detected with fluorescein-TSA, and a *Endo16* probe labeled with fluorescein and detected with Cy3-TSA. WMISH panel images were taken with a Nikon Eclipse Ti-E inverted microscope with a DS-Ri2 color CMOS camera at 20X. Nikon NIS Elements was used to acquire and crop images. Triply labeled images were taken with a Zeiss Axiovert 200M microscope, a 25x objective (Zeiss) and the Apotome system. Optical sections were stacked and cropped with Adobe Photoshop.

### 8. Microinjection of morpholino antisense oligonucleotides (MOs)

Adult sea urchins (*S*. *purpuratus*) were obtained from Point Loma Marine Invertebrate Lab (Lakeside, CA). Fertilization and microinjection was performed as described previously [44]. Morpholino-substituted oligonucleotides were from Gene-Tools (Eugene, OR). The morpholino knockdown phenotypes were confirmed by two different antisense morpholino oligonucleotides both interfering with splicing. The morpholino oligo sequences were as follows:

*SpGalnt13MO1*, CGACTCGAGTACTGAAGAGGGAGAA;
*SpGalnt13MO2*, CAAACAAACCTGTTTCTATTGATGC.

For *SpGalnt13MO1* and *SpGalnt13MO2*, the concentrations were 0.6 mM and 1.0 mM, respectively. In each case, both morpholinos gave the same phenotype, although the dose-response sometimes differed. For *SpGalnt13MO2*, the effect of interference with splicing was further confirmed by PCR of cDNAs from control and morpholino oligo injected embryos with primers on two adjacent exons (S1 Fig). Fluorescent images were taken with a Zeiss Axiovert 200M microscope, a 25x objective (Zeiss) and the Apotome system. Optical sections were stacked and cropped with Adobe Photoshop.

## Results

### Characterization of SpGalNAc-Ts

Thirteen full-length putative *S*. *purpuratus* UDP-GalNAc:Polypeptide N-Acetylgalactosaminyltransferase (SpGalNAc-T) sequences were identified, *in silico*, by screening Echinobase (http://www.echinobase.org/Echinobase/) [41]. PCR primers were then used to amplify putative orthologs from a custom- made Invitrogen cDNA library (*SpGalnt2*, *SpGalnt5*, *SpGalnt7-2*, *SpGalnt10*, *SpGalnt13*, and *SpGalnt15*) or from previously collected *S*. *purpuratus* 24-hour (*SpGalnt1*, *SpGalnt7*, *SpGalnt7-1*) 36-hour (*SpGalnt13-2*), 48-hour (*SpGalnt6* and *SpGalnt13-1*) and 24-hour-plus-48-hour (*SpGalnt11*) cDNAs.

**Fig 1:**
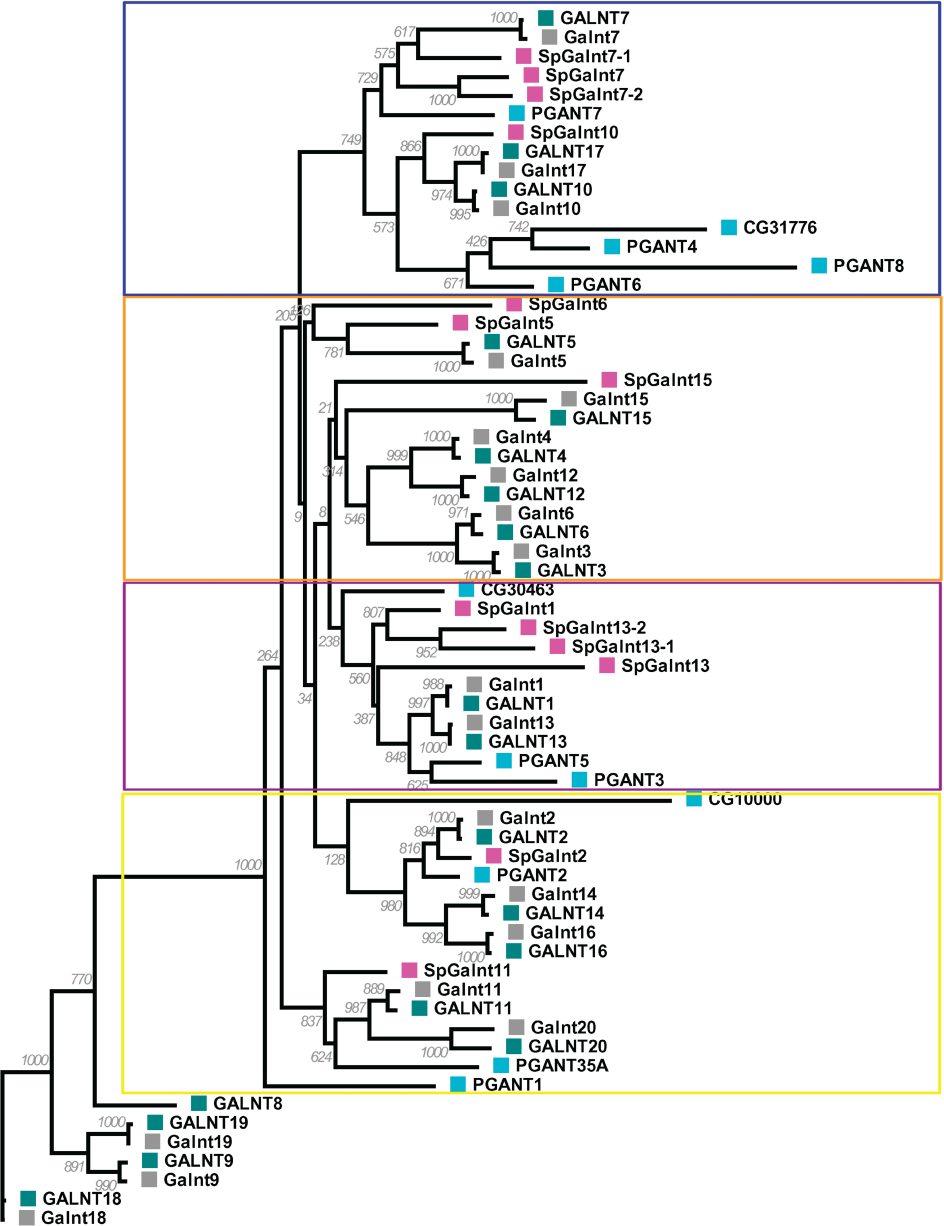
Phylogenetic tree reconstructed by maximum likelihood method, using 64 GalNAc-Ts aminoacid sequences from human (teal square), mouse (grey square), Drosophila (cyan square) and sea urchin (magenta square). Maximum likelihood bootstrap values (out of 1000 replicates) are shown next to each node.

Seven partial (*SpGalnt5*, *SpGalnt6*, *SpGalnt10*, *SpGalnt11*,*SpGalnt13-1*, *SpGalnt13-2*, and *SpGalnt15*) and six full-length (*SpGalnt1*, *SpGalnt2*, *SpGalnt7*, *SpGalnt7-1*, *SpGalnt7-2* and *SpGalnt13*) *S*. *purpuratus* GalNAc-Ts were identified. Conceptual translation of each full-length cDNA revealed characteristic features of mammalian GalNAc-Ts, (and that of a type-II membrane protein), including an N-terminal cytoplasmic region, an hydrophobic/transmembrane region, a stem region, a catalytic domain and a lectin domain. Phylogenetic trees comparing the catalytic domains of sea urchin, *Drosophila melanogaster*, mouse, and human isoforms were constructed in order to assess relatedness, using maximum-likelihood method as implemented in PhyML 3.0(Fig 1) and Bayesian inference using MrBayes software (S2 Fig). Regardless of the statistical method used, phylogenetic analyses reveal that the grouping of *SpGalnts* appears to coincide with the previously proposed mammalian GALNT gene subfamily classification [1]. For example, *SpGalnt7*, *SpGalnt7-1*, *SpGalnt7-2*, and *SpGalnt10* form one subgroup indicated by the blue box in Fig 1, consistent with the mammalian subfamily IIb (*GALNT7/T10/T17*). Similarly, *SpGalnt2* can be grouped with subfamily Ib (*GALNT2/T14/T16*) and *SpGalnt11* with subfamily If (*GALNT11/T20*) delimited by the orange box (Fig 1). Further, *SpGalnt13*, *SpGalnt13-1*, *SpGalnt13-2* and *SpGalnt1* appear to belong to the subfamily Ia (*GALNT1/T13*), demarcated by the purple box.

**Fig 2:**
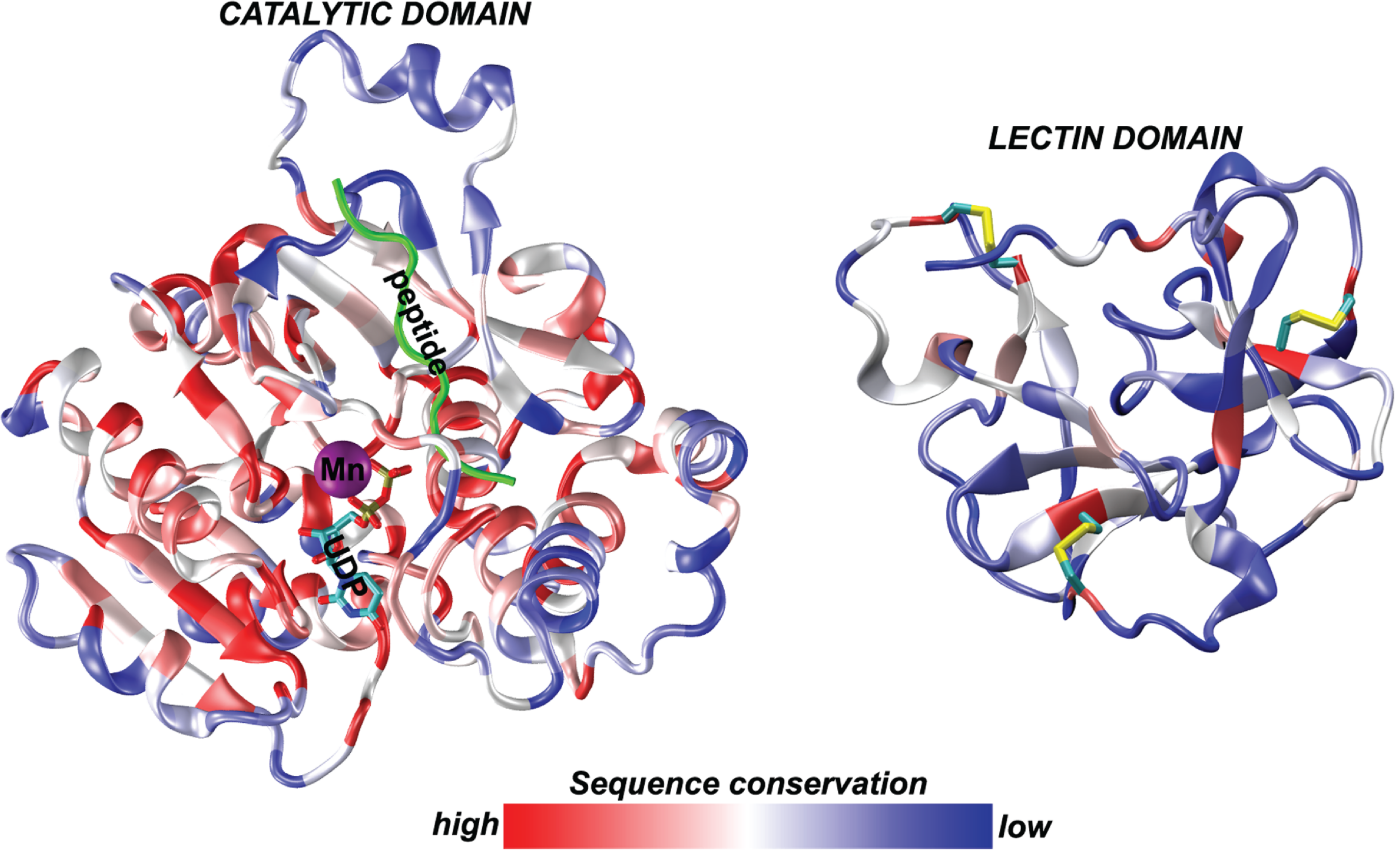
Structural representation incorporating variability data of human, mouse, and sea urchin GalNAc-T isoforms. Catalytic (left) and lectin (right) domains of human GalNAc-T2 (PDB code 2FFU), colored according to sequence conservation level, from bright red (perfectly conserved) to dark blue (highly variable). The active site of the catalytic domain contains the Mn ion (magenta sphere), a sugar donor fragment UDP (lines colored by atom type) and the acceptor peptide (green colored ribbon). Sequence variability is much higher within the lectin domain (right side), where the only conserved positions correspond to cysteine residues that form disulphide bonds which maintain structural integrity of the domain.

A sequence variability score was calculated for individual positions within the alignment of all human, mouse and sea urchin isoforms and the available structure of GALT2_HUMAN (PDB code 2FFU) was colored according to this score (Fig 2). The most highly conserved regions (shown in red) are involved either in catalysis or in maintaining structural integrity. In the catalytic domain, the central beta sheet (protein core) is very well conserved, while the peripheral helices are variable (shown in blue). Residues in contact with Mn^2+^ ion (indicated by a magenta sphere) and sugar donor (lines colored by atom type) are very well conserved, while residues interacting with the peptide (shown in green) are more variable. This sequence variability around the peptide-binding groove is thought to form the basis of isoform-specific peptide affinity within the Galnt family [3, 24], with sequence variability increasing with the distance from acceptor Ser/Thr residues in the peptide. In contrast, within the lectin domain the only perfectly conserved residues are Cys-forming disulfide bonds stabilizing the fold. All other positions exhibit much higher sequence variability (Fig 2).

### Enzymatic activity of SpGalNAc-T1, SpGalNAc-T2, SpGalNAc-T7, SpGalNAc-T7-1, and SpGalNAc-T7-2

*In vitro* enzyme assays were performed, using a panel of substrates derived from mammalian mucins [38–40]and sea urchin proteins predicted to contain O-glycans, to determine if the putative *S*. *purpuratus* isoforms represented functional transferases. The truncated coding regions of *SpGalnt1*, *SpGalnt2*, *SpGalnt7*, *SpGalnt7-1*, and *SpGalnt7-2* were inserted into a pIMKF4 expression vector and transfected into COS7 cells as previously described [37]. The secreted protein products were collected from COS7 media and FLAG-purified. As expected, we recovered recombinant SpGalNAc-T1 and -T2 in this manner. For reasons that remain unclear, three isoforms (SpGalNAc-T7, SpGalNAc-T7-1, and SpGalNAc-T7-2) were not efficiently secreted using this mammalian expression system. Therefore, we measured the enzymatic activity of these putative SpGalNAc-Ts from cell lysates.

**Fig 3:**
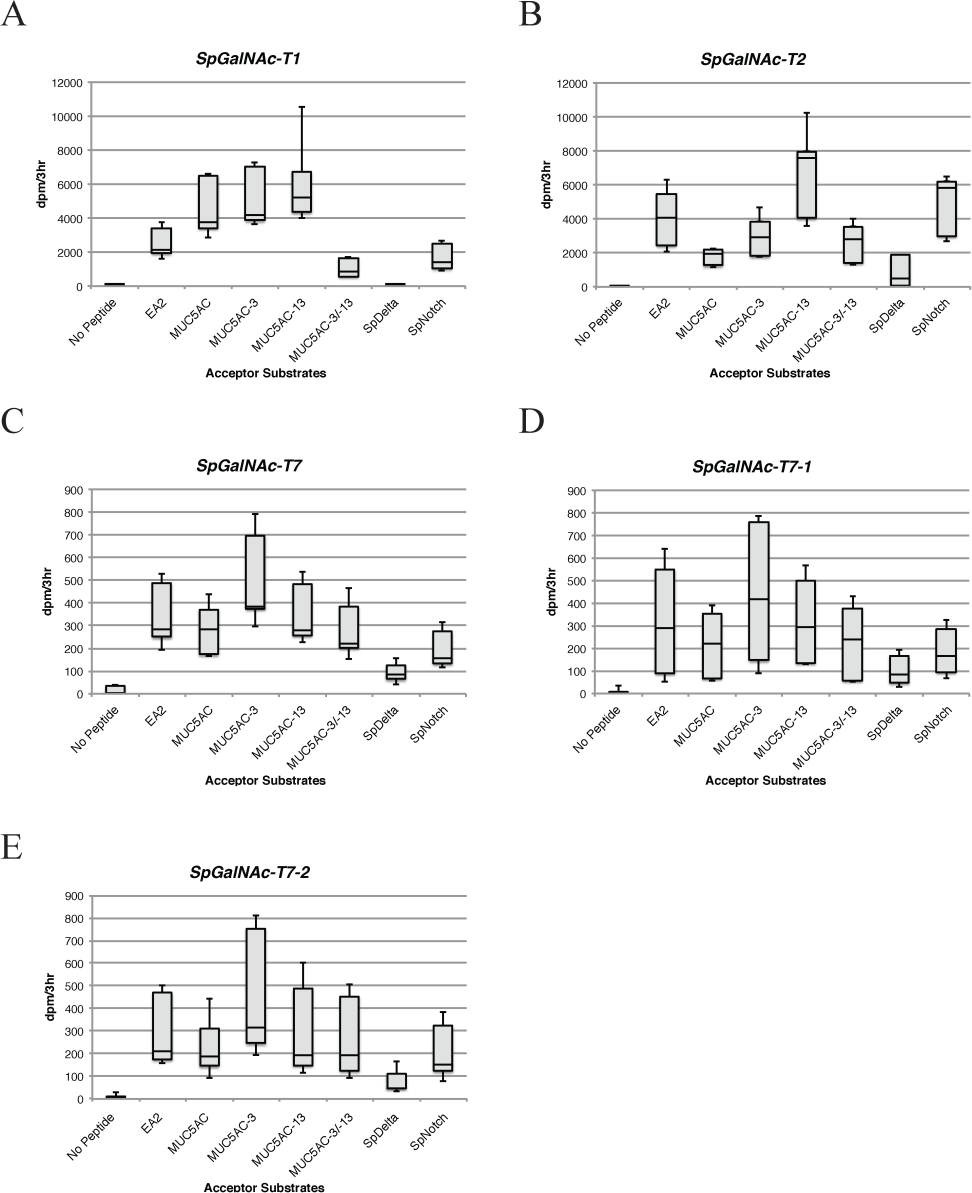
Box and whisker plots of *in vitro* enzymatic activity assays of *S*. *purpuratus* GalNAc-Ts tested against a panel of peptide and glycopeptide substrates. Comparison of activity of (A) *SpGalNAc-T1* and (B) *SpGalNAc-T2* (C) *SpGalNAc-T7* (D) *SpGalNAc-T7-1* and (E) *SpGalNAc-T7-2* using equivalent amounts of FLAG-purified protein. Each panel (A-E) shows the variability in activity (dpm/3hr) per enzyme from assays performed in triplicate from three separate transfections. Error bars indicate relative maximum and minimum activity of combined data points against each peptide tested.

GalNAc-T activity was observed for SpGalNAc-T1, SpGalNAc-T2, SpGalNAc-T7, SpGalNAc-T7-1, and SpGalNAc-T7-2 with a variety of peptides and glycopeptides whose sequences were based on either mammalian or sea urchin (Sp) substrates (Fig 3). SpGalNAc-T1 showed a preference for MUC5AC over EA2 and SpNotch (Fig 3A), whereas SpGalNAc-T2 preferred EA2 and SpNotch over MUC5AC (Fig 3B). Both SpGalNAc-T1 and -T2 also demonstrated activity against glycosylated substrates. SpGalNAc-T1 preferred monoglycosylated MUC5AC-3 or MUC5AC-13 over MUC5AC-3/-13, whereas SpGalNAc-T2 preferred MUC5AC-13 over MUC5AC-3 or MUC5AC-3/-13 (Fig 3A and 3B). Low levels of activity were observed with the SpDelta peptide for SpGalNAc-T1 and -T2 (Fig 3A and 3B).

Enzymatic activity was detected for SpGalNAc-T7 (Fig 3C), SpGalNAc-T7-1 (Fig 3D), and SpGalNAc-T7-2 (Fig 3E). However, we were unable to determine the substrate preferences of these enzymes with the panel of peptides tested in this study. These recombinant enzymes had to be enriched from cell extracts, as opposed to being produced as secreted proteins like spGalNAc-T1 and spGalNAc-T2. This results in a higher degree of variability. Additionally, the model peptide panel used in this study may not sufficiently probe the, potentially, unique substrate specificities exhibited by these enzymes.

### Structural models of SpGalNAc-T isoforms

**Fig 4:**
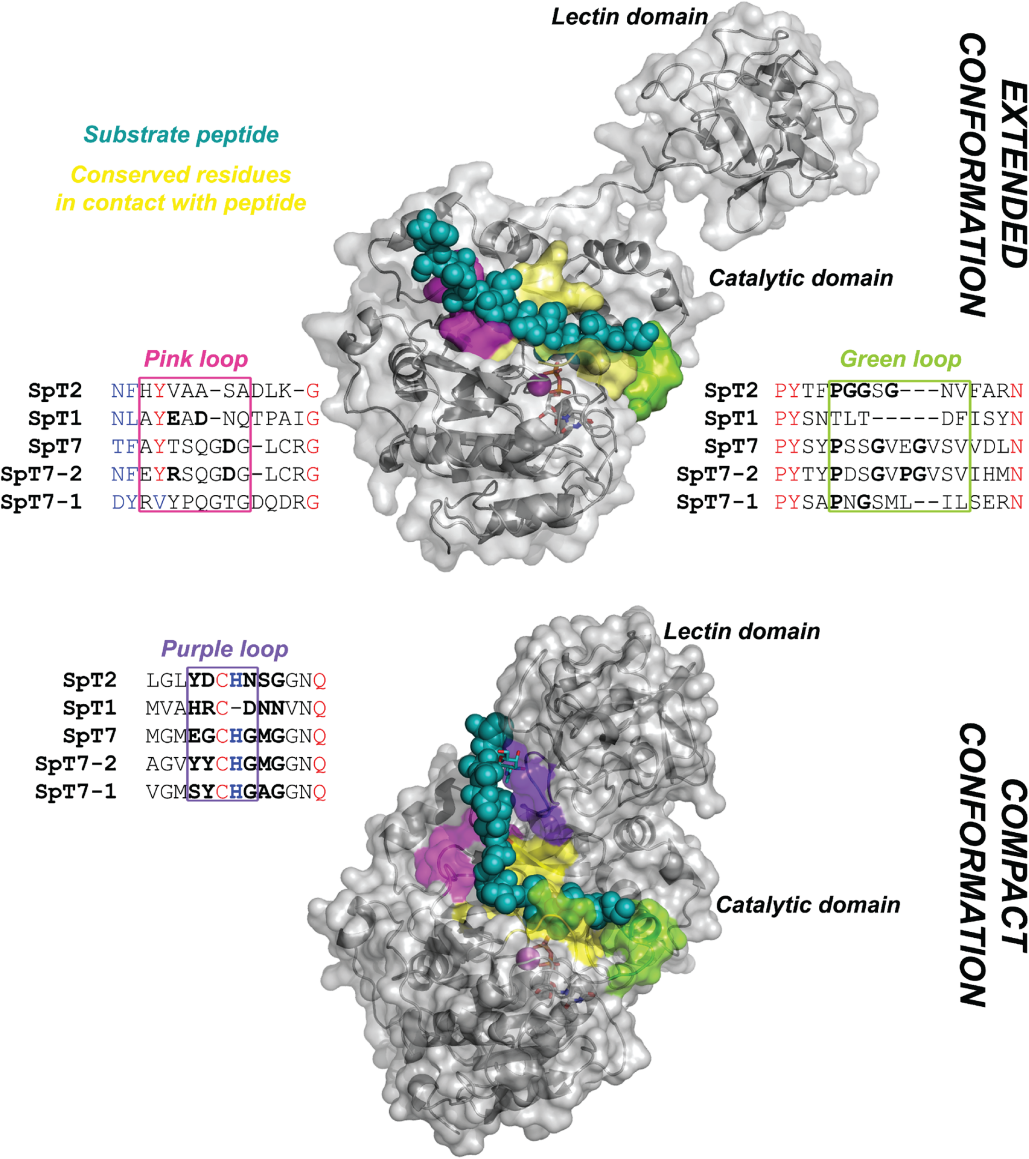
Structural mapping of enzyme sequence variability in the peptide-binding groove. Substrate peptide is depicted with dark-cyan spheres. Protein surface is colored gray, while substrate-binding groove (within 5 Å of the peptide) is colored as follows: yellow residues in the center of the groove are conserved, pink region is variable in terms of charge (sequence alignment shown in the “Pink loop” box), green region is variable in terms of flexibility (sequence alignment shown in the “Green loop” box), while the purple region indicates the residues interacting with the peptide only in the closed, compact conformation of the enzyme.

To obtain insight into the structural features that underlie the substrate preferences observed for the SpGalNAc-T isoforms, we built structural models of SpGalNAc-T1, SpGalNAc-T2, SpGalNAc-T7, SpGalNAc-T7-1 and SpGalNAc-T7-2, and identified sequence variability patterns in the enzyme peptide-binding groove (Fig 4 and S3 Fig). The central region of the peptide binding groove (surface colored yellow, Fig 4) is highly conserved, which is consistent with the highly conserved backbone structure of various peptides crystallized in complex with GalNAc-Ts in the regions flanking the glycosylation sites. In contrast, the edges of the binding groove are highly variable in terms of charge (pink color) and flexibility (green color) properties. These properties, correlated with peptides’ specific charge and flexibility distributions, likely explain peptide substrate preferences, as observed previously by Gerken et al.[3].

The increased negative charge of SpGalNac-T1 isoform in the “pink loop” edge (Fig 4) (due to the presence of two negative charges and no positive charges) compared to other isoforms may correlate with its lowest affinity towards SpNotch peptide, carrying the highest negative charge among tested peptides.

For the enzyme-substrate interaction to occur, the peptide substrate requires specific flexibility patterns in the enzyme binding groove, most likely located in the “Green loop” edge (Fig 4). Loop conformation is guided by a conserved Pro residue (found in all isoforms except SpGalNAc-T1), with further flexibility being dictated by the number of Gly residues. The SpGalNAc-T2 “Green loop” is most flexible due to the presence of three Gly residues and it displays preference for the rigid SpNotch peptide, which contains three Pro residues. Other isoforms which contain one or two Gly residues have lower activity against SpNotch. In the case of SpGalNAc-T1, the “Green loop” lacks both the Pro residue conserved in other isoforms and also any Gly residues. This likely results in a more rigid conformation in the loop region, and may explain why the activity against SpNotch peptide is very low.

### Temporal and spatial expression of *SpGalnt* transcripts

**Fig 5:**
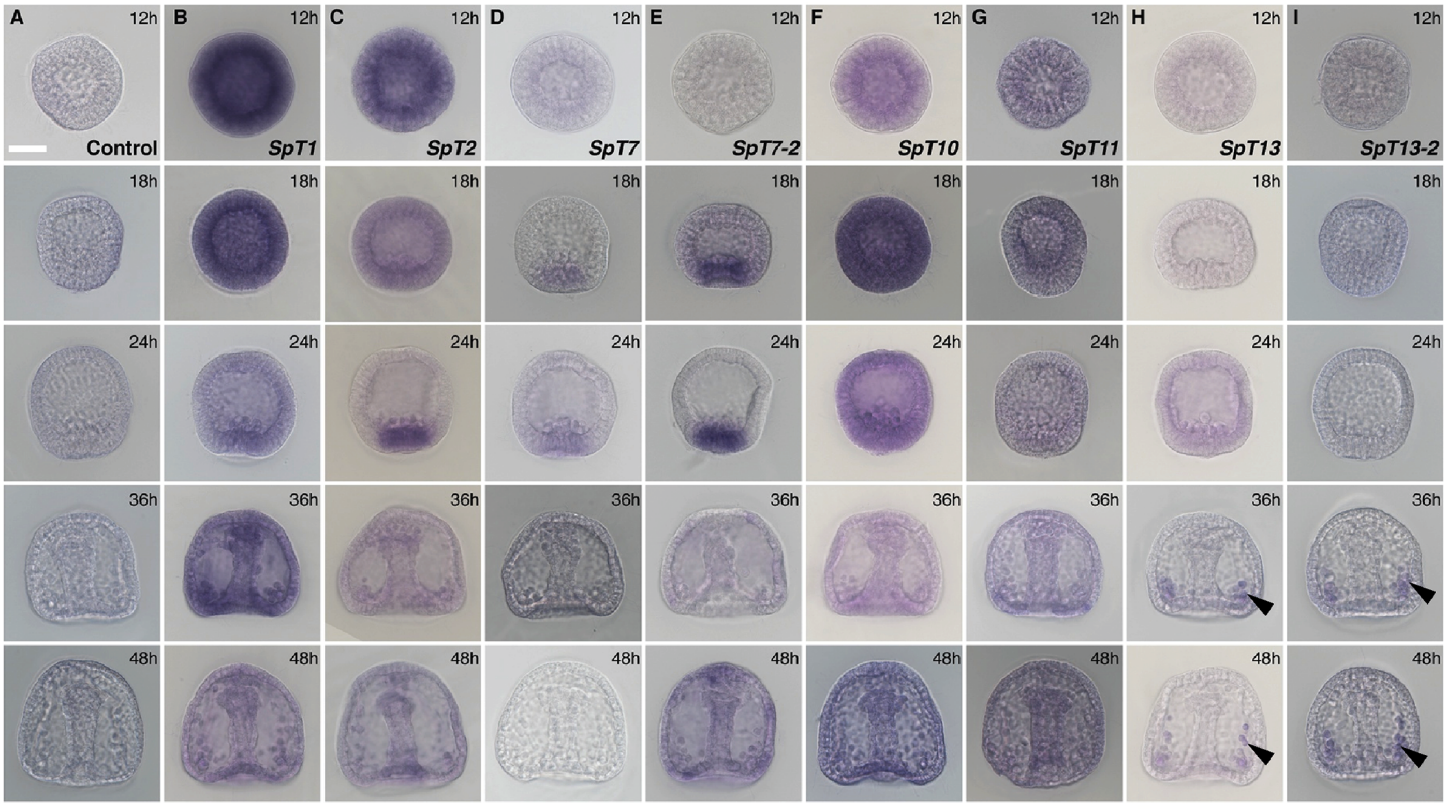
Spatial and temporal expression of *SpGalnt*s in developing sea urchin embryos. Whole-mount mRNA *in situ* hybridizations of *SpGalnt1* (B), *SpGalnt2* (C), *SpGalnt7* (D), *SpGalnt7-2* (E), *SpGalnt10* (F) *SpGalnt11* (G), *SpGalnt13* (H) and *SpGalnt13-2* (I) at 12, 18, 24, 36 and 48 hours post fertilization (hpf). In column (A), a sense *SpGalnt7-1* probe was used as negative control. All representative embryos of three separate experiments are shown in the lateral view. Scale bar in (A) indicates 20 μm.

Whole-mount RNA *in situ* hybridization was used to determine spatial and temporal expression of sea urchin GalNAc-T transcripts in early development. Digoxigenin-labeled RNA probes were used to observe the spatial expression patterns of genes at 12 hpf (early blastula), 18 hpf (hatching blastula), 24 hpf (mesenchyme blastula), 36 hpf (gastrula), and 48 hpf (late gastrula) (Fig 5) (see fate map in S4 Fig). To optimize comparisons among stages, hybridizations for a given GalNAc-T were carried out in the same reaction, i.e. with the same probe for the same hybridization time and histochemical reaction time. No signal was detectable by this method for four of the putative (*SpGalnt5*, *SpGalnt6*, *SpGalnt13-1*, and *SpGalnt15)*, and one of the functional (*SpGalnt7-1)* sea urchin isoforms at the developmental stages tested (data not shown). The remaining eight isoforms exhibited diverse patterns of mRNA expression. Transcripts of *SpGalnt1*, *SpGalnt2*, and the putative isoforms *SpGalnt10* and *SpGalnt11* were uniformly distributed at early blastula stages. As shown in Fig 5, column B, *SpGalnt1* expression was biphasic, with signals decreasing from early blastula to mesenchyme blastula stage (24hr), followed by uniform re-accumulation throughout gastrulation (36 h and 48 h). *SpGalnt2* levels showed a similar early decrease in most regions of the embryo. Higher levels persisted transiently in endomesoderm at late blastula stages (24 h), after which signals decreased to low-level ubiquitous expression (cf Fig 5, column C vs. column A or column D. 48 hr). *SpGalnt7* was expressed transiently at 18 h and 24 h with low levels detectable only in endomesoderm (Fig 5, column D); transcripts of its more highly expressed close relative *SpGalnt7-2* also accumulated at both 18 h and 24 h blastula stages in a similar region of the vegetal plate, followed by a low level of ubiquitous expression (Fig 5, column E) (Transcriptome analysis indicates that transcript levels of SpGalnt7 and SpGalnt7-2 peak at 700 (24 hpf) and 5000 (18 hpf) transcripts/embryo, respectively [45]). As shown in Fig 5, column F, putative *SpGalnt10* was ubiquitously expressed throughout development with peak accumulation occurring prior to gastrulation at 18 h and 24 h. Putative *SpGalnt11* probes gave only very low signals, with no detectable enrichment in any region or at any stage examined (Fig 5, column G). The most highly restricted expression was observed for putative isoform *SpGalnt13* and *SpGalnt13-2* transcripts, which were detected only in the skeletogenic mesenchyme cells after gastrulation (Fig 5, columns H and I; arrows in 36 h and 48h).

**Fig 6:**
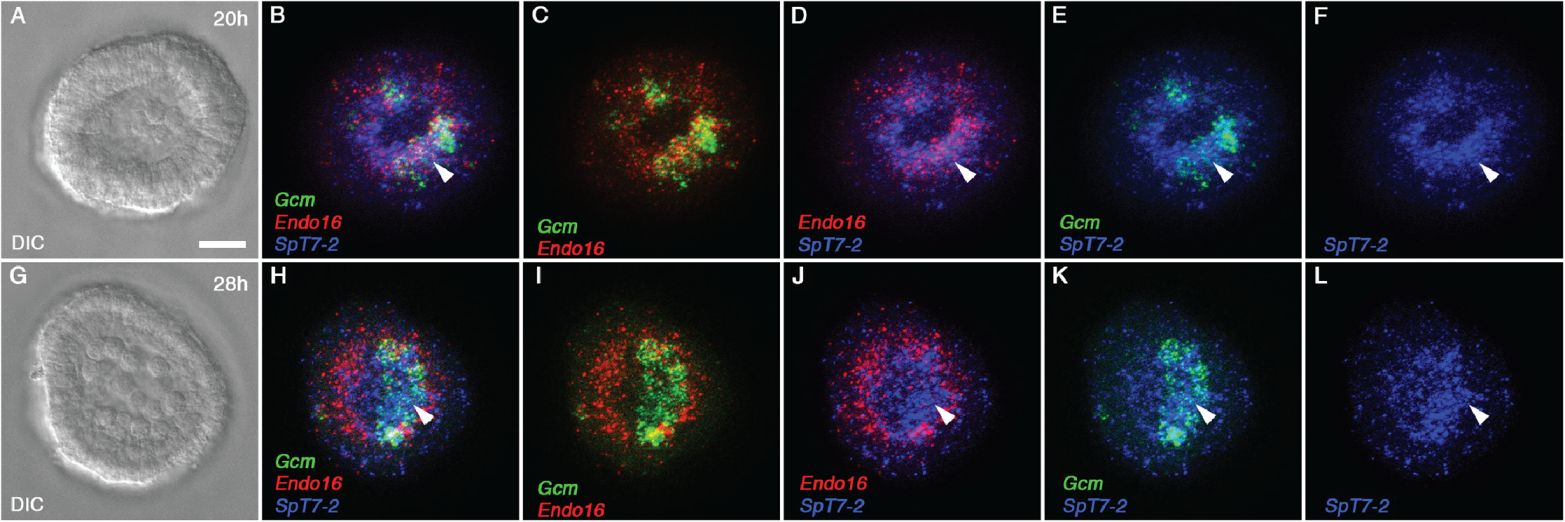
*SpGalnt7-2* is expressed in endomesoderm cells in mesenchyme blastula stage sea urchin embryos. Triple fluorescence *in situ* hybridization of *SpGalnt7-2* (blue), *Gcm* (green), a secondary mesenchyme gene marker, and *Endo16* (red) an endoderm marker in representative sea urchin embryos at 20 hpf and 28 hpf. All embryos are shown in vegetal view as depicted by DIC images (A) and (G). (B-E) triple and double fluorescence channel images of a 20 hpf embryo. (H-K) triple and double fluorescence channel images of a 28 hpf embryo. Arrows in (B), (D), and (E) indicate the co-localization of *SpT7-2* with *Gcm* and *Endo16* in the endomesoderm at 20 hpf. Arrows in (H), (J), (K) and (L) show a shift in *SpGalnt7-2* expression to the aboral side in secondary mesenchyme cells at 28 hpf. Panels (F) and (L) show *SpGalnt7-2* expression only. All representative embryos of three separate experiments are shown in the vegetal view. Scale bar in (A) indicates 20 μm.

To better define the tissues giving the strongest signals in endomesoderm, *SpGalnt7-2*, a triple fluorescent *in situ* hybridization (FISH) was performed to compare their distribution to those of two canonical markers, *Endo16* and *Gcm*. At 20 h Endo16 is expressed throughout the ring of endomesoderm cells surrounding a central disc of unlabeled cells that includes primary mesenchyme and the small micromeres; Gcm is expressed only in a subset of cells that give rise to secondary mesenchyme. [46, 47]. At 20 hpf, the *SpGalnt7-2* signal overlaps with those for both *Gcm Endo16* and *Gcm*, (e.g. arrows in Fig 6B, 6D and 6E). At 28 hpf, *Endo16* expression remains uniform throughout the torus of endomesoderm (Fig 6J), while *Gcm* expression shifts to form a crescent on the future aboral side [47], Fig 6I). *SpGalnt7-2* expression in 28 h mesenchyme blastulae (Fig 6L) overlapped with the crescent (Fig 6H and 6K). Therefore, as development progresses it appears that *SpGalnt7-2* may play early roles in both endoderm and secondary mesenchyme and a continuing role in secondary mesenchyme.

### Morpholino oligonucleotide-mediated knockdown of SpGalnt13 transcripts results in deficiencies in embryonic skeleton and neurons

**Fig 7:**
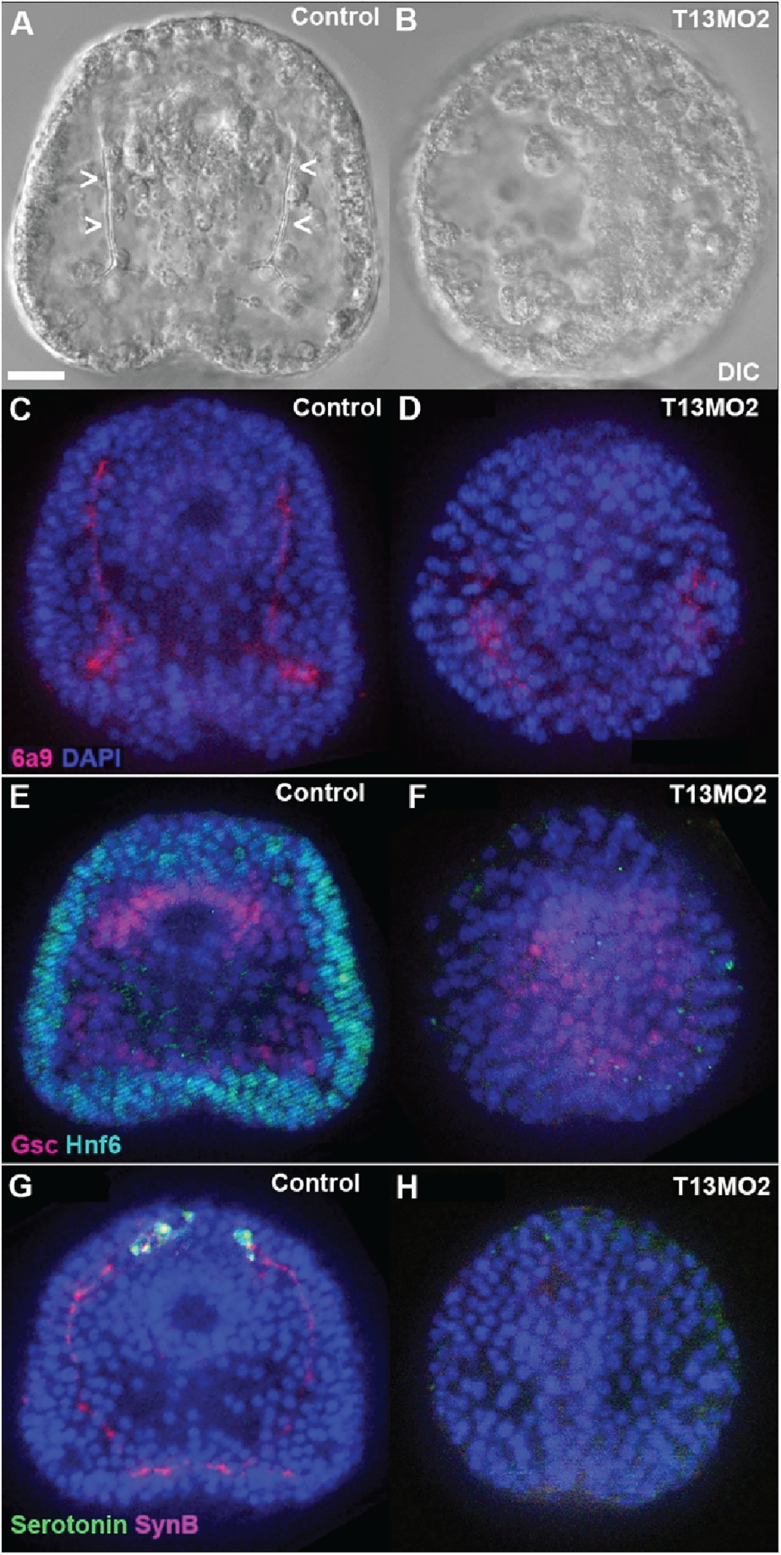
*SpGalNAc-T13* is required for embryo skeleton and nerve cell development. (A-B) DIC images of 3-day embryos showing skeletal spicules in the control embryo (A, arrowheads) compared to a lack of spicules in the *SpGalnt13* morphant (B). (C-H) fluorescent images of expression patterns of different markers. (C-D) Primary mesenchyme cell (PMC) antibody marker 6a9 (red) was detected in both control embryos (C) and *SpGalnt13* morphants (D), indicating that PMC cells ingressed in the morphant but could not form spicules. (E-F) Oral ectoderm marker Goosecoid (GSC, red) was detected in both the control embryo (E) and the *SpGalnt13* morphant (F), but ciliated band ectoderm marker Hnf6 (green) was not detected in the *SpGalnt13* morphant. (G-H) Serotonergic neuron marker (green) and pan-neuronal marker Synaptotagmin B (SynB, red) were detected in the control embryo (G) but absent in the *SpGalnt13* morphant (H). All embryos are shown in oral view, with the anterior pole to the top. Scale bar in (A) indicates 20 μm. Representative embryos are shown from three separate experiments.

As a first test of the developmental function of SpGalNAc-Ts in developing sea urchin embryos, microinjection of morpholino antisense oligonucleotides (MOs) into fertilized eggs was carried out to specifically inhibit the splicing of targeted *SpGalnt13*. These embryos exhibited multiple specific defects. First, they failed to form the skeletal spicules that support the larva’s pyramidal shape as it undergoes morphogenesis from prism to pluteus (Fig 7A–7D). This result is consistent with the *in situ* hybridization demonstration (Fig 5, column H), of *SpGalnt13* transcript accumulation in skeletogenic mesenchyme cells, beginning after gastrulation. Interestingly, individual cells in the blastocoels of morphants did stain with the 6a9 early PMC-specific antibody, as shown in Fig 7C and 7D. By the criterion of 6a9 expression, PMCs are specified and they ingress into the blastocoel of morphants, but are blocked in the later differentiated function of spiculogenesis.

*SpGalnt13* was also found to play a role in ectoderm development. *SpGalnt13* morphants correctly established oral-aboral polarity, as shown by expression of the oral marker goosecoid (Gsc) (Fig 7E and 7F, red) and the aboral ectoderm marker Spec1 (S5A and S5B Fig, green) on opposite sides of the embryo. In addition, pigment cells (a secondary mesenchyme derivative) differentiated and migrated to the aboral ectoderm as in control embryos (S5C and S5D Fig red). However, as shown by staining for the specific marker Hnf6 (green, Fig 7F), *SpGalnt13* morphants failed to differentiate a ciliary band between the oral and aboral territories. Reflecting the lack of the constricting ciliary band and absence of skeletal spicules, *SpGalnt13* morphants adopted a rounded appearance at pluteus stage (S5 Fig). Since neural cells differentiate in, or adjacent to, the ciliated band, we further tested for the formation of neurons using antibodies that recognize synaptotagmin B, which is expressed by all neurons (synB, red) or serotonin (green) which, at early pluteus stage, is expressed only by a few neurons in the apical plate. Surprisingly, signals for both neural markers were eliminated in *SpGalnt13* morphants (Fig 7G and 7H), suggesting that SpGalNAc-T13 also is required for proper neural development. A similar phenotype was observed with a second splice-blocking morpholino (S6 Fig).

## Discussion

We present the first characterization of the GalNAc-T family in embryos of the sea urchin, *S*. *purpuratus*. A total of 13 putative isoforms were identified from the sea urchin genome. A detailed phylogenetic analysis comparing *Drosophila*, sea urchin, mouse and human isoforms indicated high levels of sequence conservation between sea urchin and mammalian species, while *Drosophila* isoforms appear more divergent. Furthermore, we found the sea urchin isoforms to be grouped into subfamilies consistent with the *GALNT* gene family classification proposed previously [1]. As expected, the structural representation incorporating variability data of human, mouse and sea urchin GalNAc-Ts displayed the highest levels of similarity within the catalytic domain. In contrast, the lectin domain exhibits high levels of sequence variability with conservation only among the Cys residues that maintain domain structural integrity. Sequence comparison of lectin domains between mouse and human isoforms exhibit significantly greater levels of identity (~95%) than identity levels found between sea urchin and either mouse or human lectin domains (<50%) (data not shown). Previous studies show that the lectin domain functions to modulate substrate specificity and improve GalNAc-T catalysis [3, 48, 49]. Therefore, high levels of lectin domain variability among sea urchin and mammalian isoforms suggest that sea urchin lectin domains may be recognizing very different substrates than their mammalian counterparts. Preliminary profiling of O-linked oligosaccharides found in 24 hour sea urchin embryos revealed the presence of a predominant sulphated core 1 glycan (data not shown); it remains to be determined if this structure is preferentially recognized by the lectin domains of the SpGalNAc-Ts.

Transcripts of eight of the thirteen functional or putative GalNAc-Ts were detectable by whole-mount in situ hybridization, revealing that expression of family members is highly regulated temporally and/or spatially (Fig 5). In all cases but *SpGalnt13*, *SpGalnt13-1* and *SpGalnt13-2*, highest expression is observed before gastrulation, when cell division and specification of major tissue areas are occurring in the absence of growth, and the expression domain of each GalNAc-T encompasses all (e.g. *SpGalnt1*, *SpGalnt2* and *SpGalnt10*) or many presumptive cell types. For example, the spatially restricted expression domains of *SpGalnt2*, *SpGalnt7* and *SpGalnt7-2* include the precursors to a tripartite gut with differentiated pharynx, midgut and hindgut separated by myoepithelial sphincters and at least 4 distinct secondary mesenchyme cell types. As we showed by triple-labelled in situ hybridization, *SpGalnt7-2* expression initially is uniform throughout the vegetal plate and subsequently selectively downregulated in future endoderm while persisting in some secondary mesenchyme. Preliminary tests of *SpGalnt7-2* function by morpholino-mediated knockdown showed only delayed development of the archenteron/gut, likely reflecting partially redundant function of the several SpGalnts expressed in endomesoderm (data not shown).

In contrast to the broad expression patterns of the most GalNAc-Ts, SpGalNAc-T13 and SpGalNAc-T13-2 showed, by *in situ* hybridization, apparent restriction to a single cell type beginning at a relatively late point in its development (Fig 5H and 5I). Transcripts were detectable only in skeletogenic mesenchyme after gastrulation, i.e. only after the PMC precursors have been specified (i.e. expressed the specific 6a9 marker), ingressed into the blastocoel, and taken up specific positions on the blastocoel walls, where they normally fuse to form characteristic syncytial cables that dictate the architecture of the endoskeleton [50, 51]. All of these initial events appeared to occur normally in morphants, but the cells failed to secrete the calcite skeletal rods (Fig 7). This observation implicates SpGalNAc-T13 in processes that could include secretion of the large number of spicule matrix proteins, and/or calcite deposition. There are precedents for members of this enzyme family influencing protein secretion in both mammals [6] and *Drosophila* [52]. Several skeletogenic proteins are secreted during development by the primary mesenchyme cells [53]. One of these, SpSM30B/C, has 6 putative O-glycosylation sites [54]. However, it remains to be established if glycosylation of this protein has any functional role in secretion or spiculogenesis.

An initially surprising result was the failure of *SpGalnt13* morphants to differentiate a ciliary band and neurons. One possible formal explanation for this observation would be that skeletogenic mesenchyme cells have a signaling function required for aspects of ectoderm differentiation including specification of neurons and ciliary band. A more likely possibility is suggested by quantitative analysis of *SpGalnt13* transcript levels during development [45], which shows that they accumulate sharply between 18hr and 24hr of development to approximately 800 copies per embryo, and then diminish in abundance only slightly through gastrula stages. Transcript concentrations of 10–20 copies per skeletogenic mesenchyme cell were detected by in situ hybridization, i.e. 800 transcripts in ~ 50 skeletogenic mesenchyme cells. However, if *SpGalnt13* initially is broadly expressed in the early embryo, then the levels of ~1 copy per equivalent cell volume would not be detectable.

Studies of glycosyltransferase function, as assessed by gene knockdown experiments, are often difficult to evaluate since glycosyltransferases may have multiple substrates and therefore, unlike studies of signaling pathways and transcription factors in gene regulatory networks, glycosyltransferases are likely to influence multiple pathways involved in development as well as proteins involved in differentiated functions of various cell types. A further complication in assessing glycosyltransferase function is that we do not know the relationship of transcript level to enzyme activity, the later a reflection of enzyme persistence and substrate specificity. Despite these limitations it is clear from the current work that SpGalNAc-Ts play essential roles in early sea urchin development.

## Supporting information

S1 Fig *SpGalnt13MO2* blocked splicing in injected embryos. PCR results of cDNAs from control (middle lane) and morpholino injected (right lane) embryos showed that the 269bp expected band was reduced. Instead, an unspliced band of 963bp was detectable. The PCR Primer sequences are GCAGCAAGTCGTAATGCTAC/ GCAGGATGCTGCAACACCA.
(TIF)

S2 Fig Phylogram of the same 64 sequences as in Fig 1 obtained from Bayesian inference using MrBayes software. Values next to each node correspond to the Bayesian posterior probability (BPP).
(TIF)

S3 Fig Structure-based sequence alignment of human GalNAc-T2 (5AJP) and SpGalNAc-T isoforms. Identical residues are colored red, similar residues are colored blue. Yellow highlight indicates conserved residues situated within 5A of the substrate peptide, while colored frames (pink, green and purple) correspond to variable loops within 5A of the peptide (same color code as in Fig 4). Purple loop interacts with the peptide only in the closed, compact conformation of the enzyme, when lectin domain is in vicinity of the catalytic domain.
(TIF)

S4 Fig **Fate map of mesenchyme blastula (A: lateral view; C: vegetal view), late gastrula (B: lateral view) and pluteus (D: left, oral view; right, lateral view) stage embryos.** Primary mesenchyme cells (red) initiate vegetal plate ingression followed by secondary mesenchyme cells (pink) and endoderm (yellow). Ectoderm (blue) surrounds presumptive endoderm and mesodermal cell types at larval stages. White bars in D represent the skeleton.
(TIF)

S5 Fig *SpGalNAc-T13* is not required for oral-aboral polarity or pigment cell development. Optical slices of 3-day control and *SpGalnt13* morpholino-injected embryos. (A-B) Polarized distribution of the aboral ectoderm marker (Spec1, green) was detected in both the control (A) and the *SpGalnt13* morphant (B) showing that *SpGalnt13* knockdown does not disrupt oral/aboral polarity. (C-D) Development of pigment cells appeared as shown by staining for the pigment cell marker (Sp1, red) in both control (C) and *SpGalnt13* morphants (D). Representative embryos from two separate experiments are shown in lateral view.
(TIF)

S6 Fig *SpGalnt13MO1* has the same effect on embryonic development as that of *SpGalnt13MO2* (Fig 7). **(A and B)** DIC images of **c**ontrol (A) and *SpGalnt13MO1-* (B) injected embryos, showing the absence of spicules (arrow heads in A) in the morphant. (C and D) The ciliated band marker Hnf6 (green in control embryo C) was not detectable in the morphant (D), but the PMC marker 6a9 was detected in both control and morphant. (E and F) control (E) and morphant (F) embryos stained with antibodies to serotonin (green) and synB (red) show that both neural signals were greatly reduced in the morphant. Embryos are shown in oral view and the animal pole is to the top. The white bar in A represents 20 μm.
(TIF)
