# Supplementary figures and images for: Characterization and expression analysis of Galnts in developing Strongylocentrotus purpuratus embryos
Source: PLoS One. 2017 Apr 27;12(4):e0176479. doi: 10.1371/journal.pone.0176479 (PMC5407767; doi:10.1371/journal.pone.0176479)

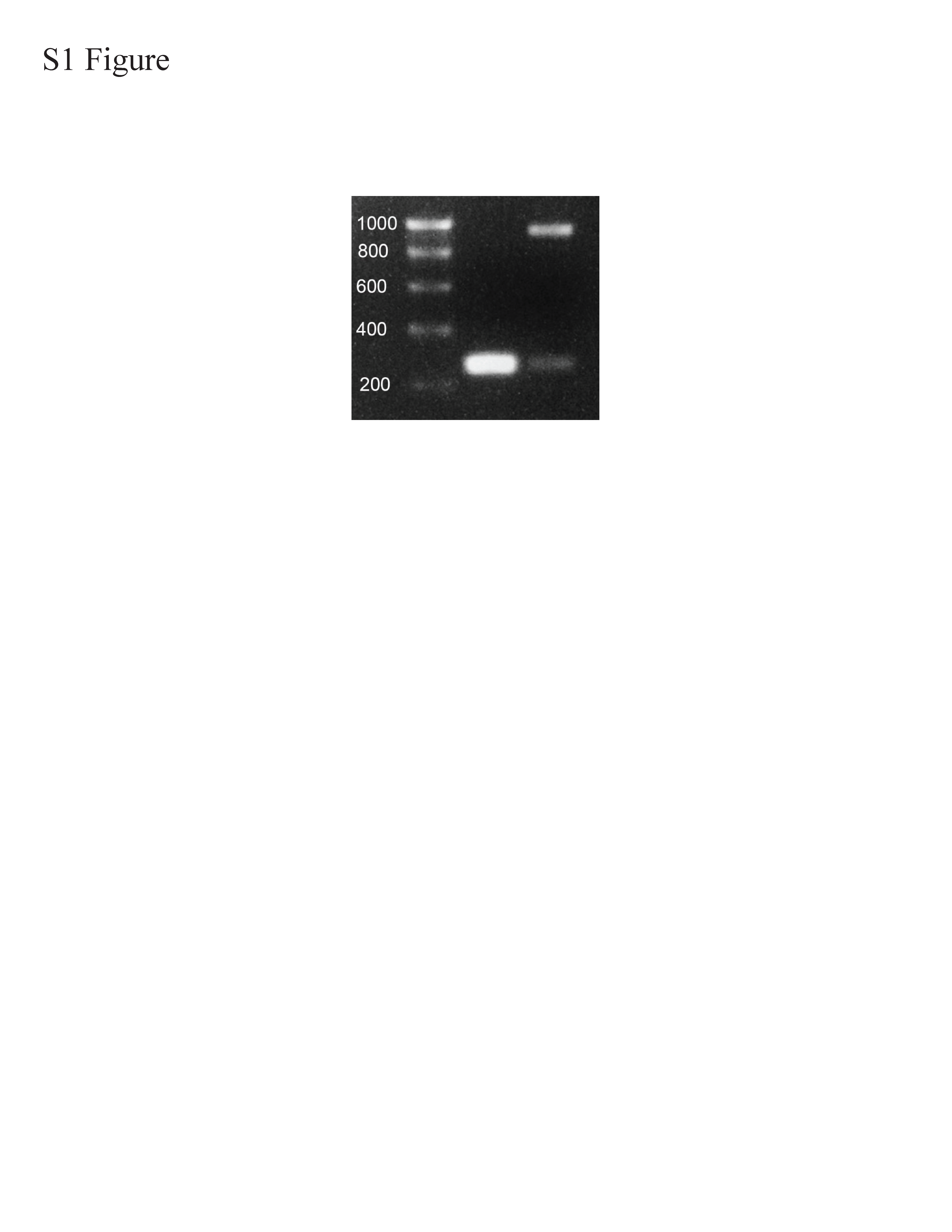

Supplement: S1 Fig — PCR results of cDNAs from control (middle lane) and morpholino injected (right lane) embryos showed that the 269bp expected band was reduced. Instead, an unspliced band of 963bp was detectable. The PCR Primer sequences are GCAGCAAGTCGTAATGCTAC/ GCAGGATGCTGCAACACCA. (TIF) [file pone.0176479.s001.tif]

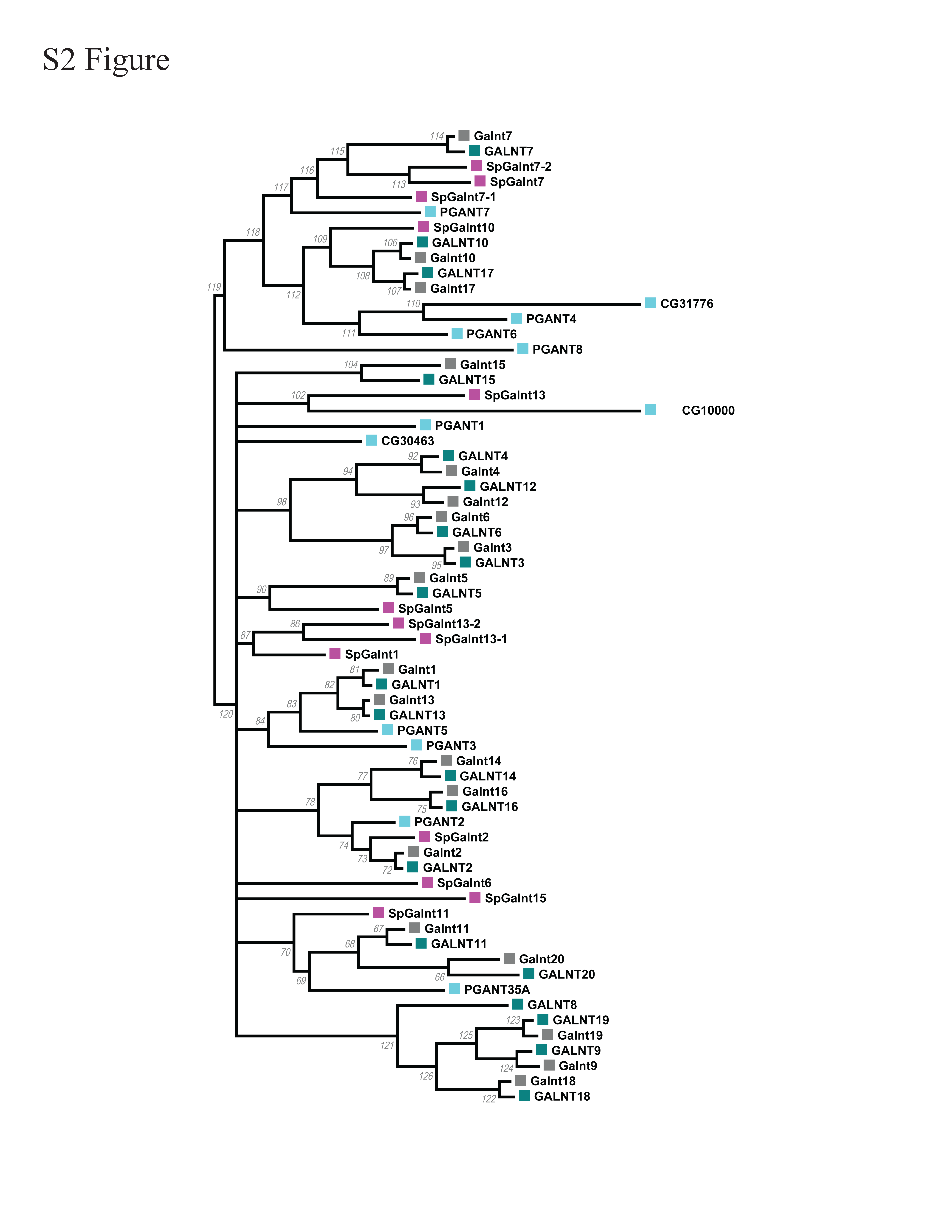

Supplement: S2 Fig — Values next to each node correspond to the Bayesian posterior probability (BPP). (TIF) [file pone.0176479.s002.tif]

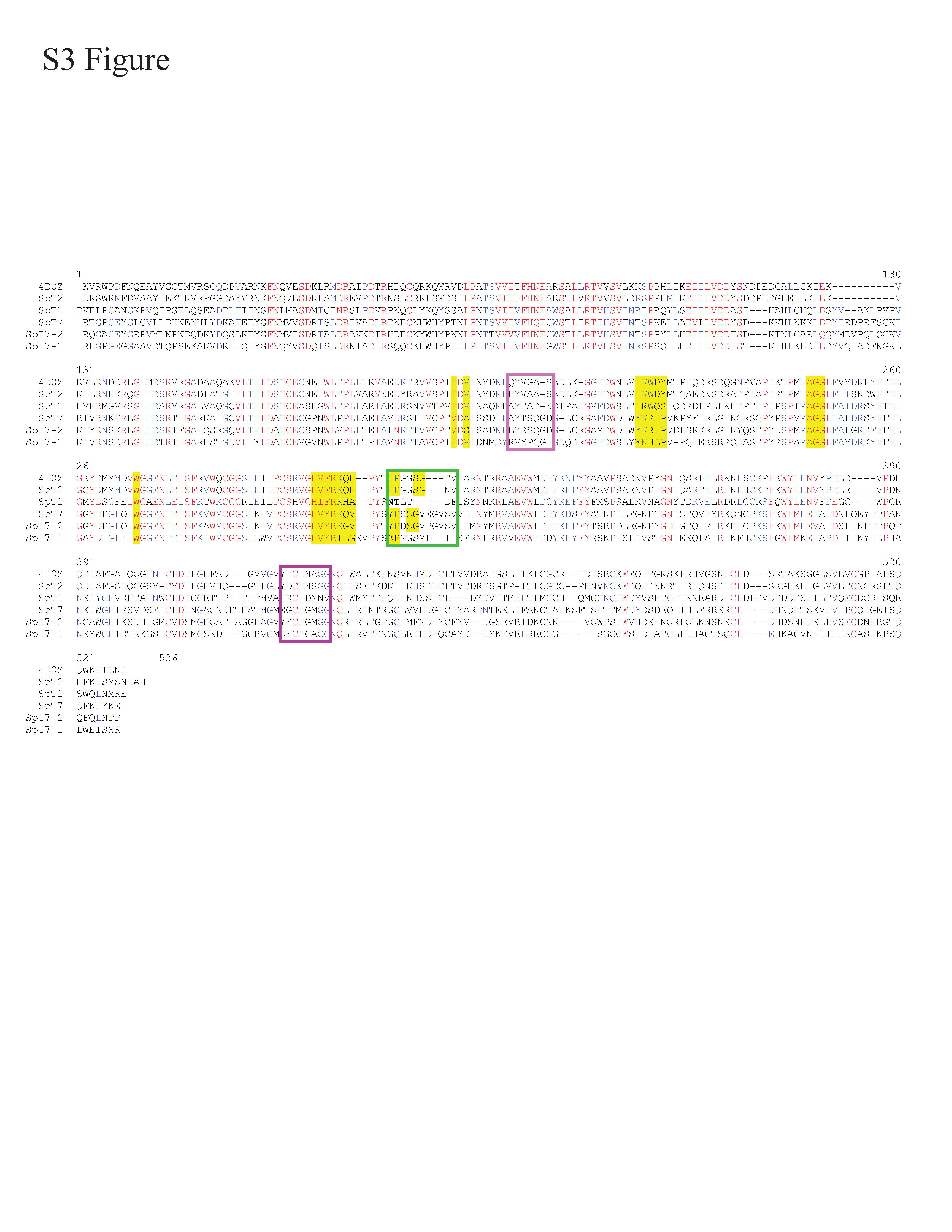

Supplement: S3 Fig — Identical residues are colored red, similar residues are colored blue. Yellow highlight indicates conserved residues situated within 5A of the substrate peptide, while colored frames (pink, green and purple) correspond to variable loops within 5A of the peptide (same color code as in Fig 4). Purple loop interacts with the peptide only in the closed, compact conformation of the enzyme, when lectin domain is in vicinity of the catalytic domain. (TIF) [file pone.0176479.s003.tif]

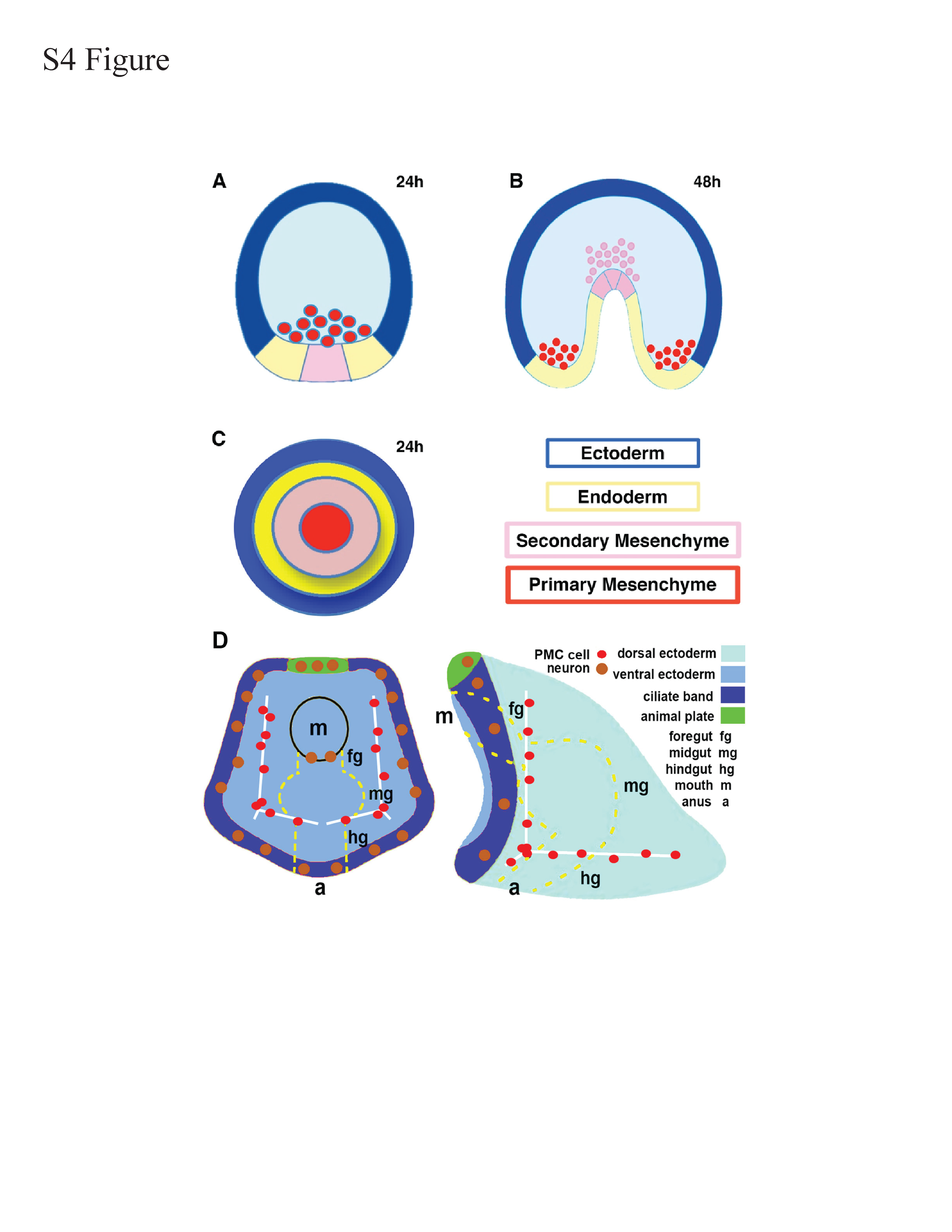

Supplement: S4 Fig — Fate map of mesenchyme blastula (A: lateral view; C: vegetal view), late gastrula (B: lateral view) and pluteus (D: left, oral view; right, lateral view) stage embryos. Primary mesenchyme cells (red) initiate vegetal plate ingression followed by secondary mesenchyme cells (pink) and endoderm (yellow). Ectoderm (blue) surrounds presumptive endoderm and mesodermal cell types at larval stages. White bars in D represent the skeleton. (TIF) [file pone.0176479.s004.tif]

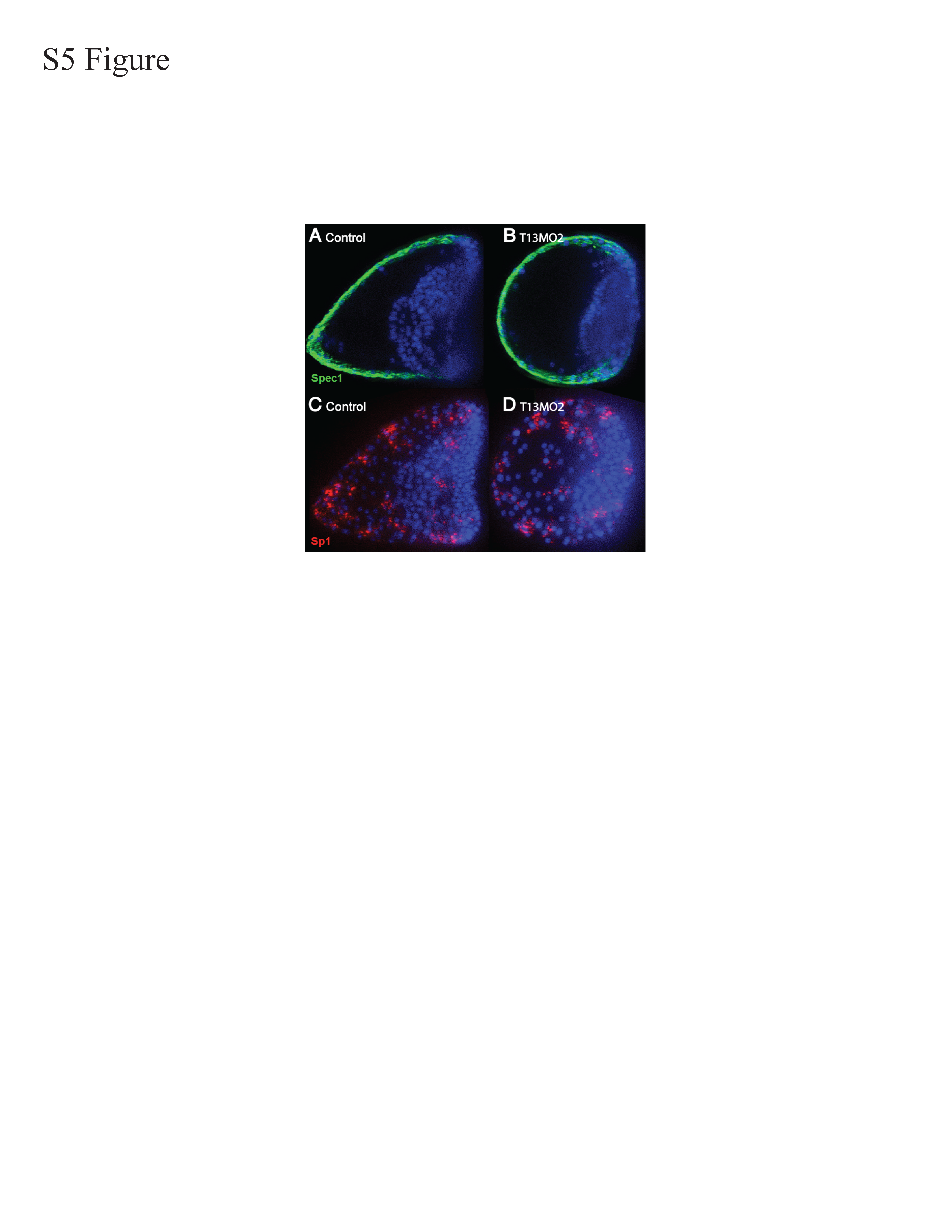

Supplement: S5 Fig — Optical slices of 3-day control and SpGalnt13 morpholino-injected embryos. (A-B) Polarized distribution of the aboral ectoderm marker (Spec1, green) was detected in both the control (A) and the SpGalnt13 morphant (B) showing that SpGalnt13 knockdown does not disrupt oral/aboral polarity. (C-D) Development of pigment cells appeared as shown by staining for the pigment cell marker (Sp1, red) in both control (C) and SpGalnt13 morphants (D). Representative embryos from two separate experiments are shown in lateral view. (TIF) [file pone.0176479.s005.tif]

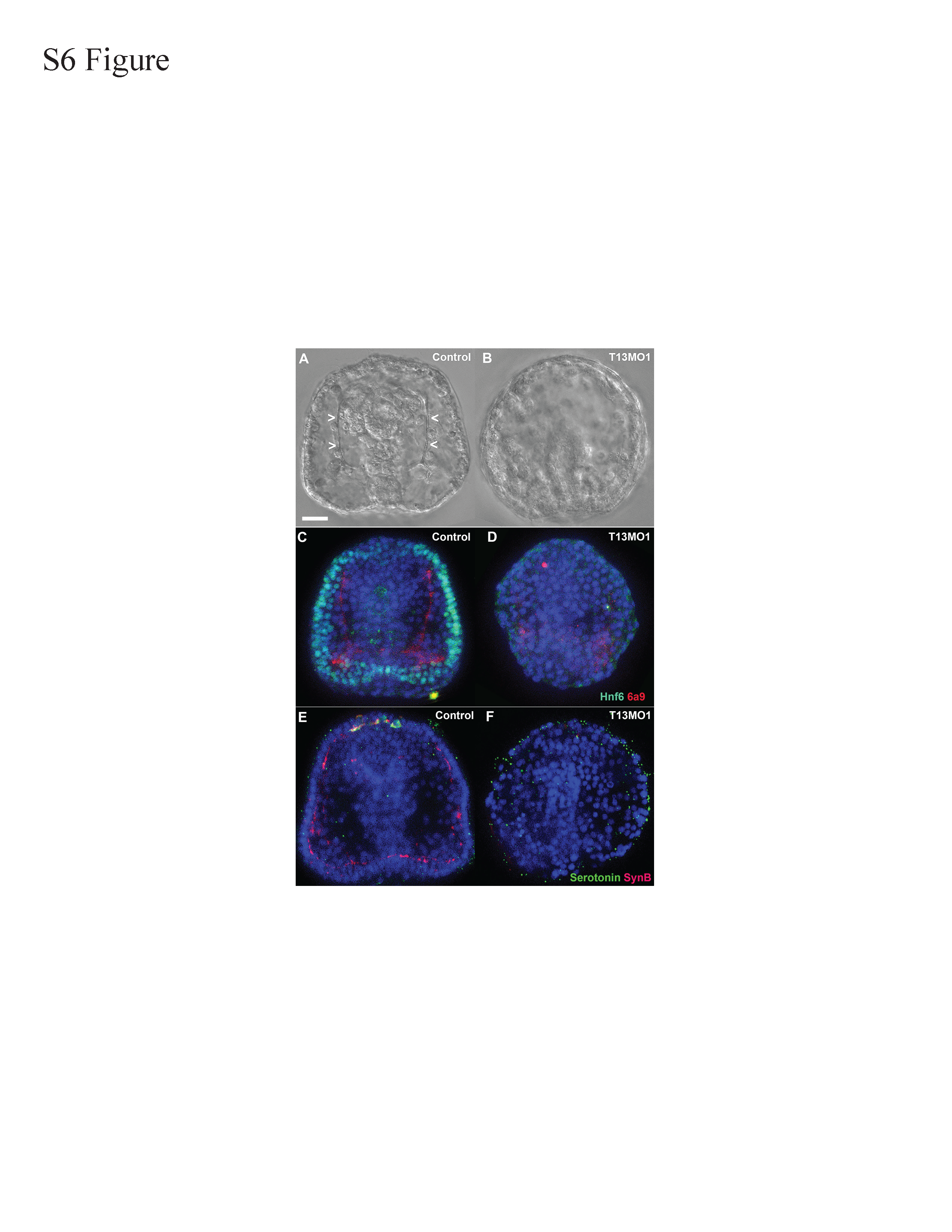

Supplement: S6 Fig — (A and B) DIC images of control (A) and SpGalnt13MO1- (B) injected embryos, showing the absence of spicules (arrow heads in A) in the morphant. (C and D) The ciliated band marker Hnf6 (green in control embryo C) was not detectable in the morphant (D), but the PMC marker 6a9 was detected in both control and morphant. (E and F) control (E) and morphant (F) embryos stained with antibodies to serotonin (green) and synB (red) show that both neural signals were greatly reduced in the morphant. Embryos are shown in oral view and the animal pole is to the top. The white bar in A represents 20 μm. (TIF) [file pone.0176479.s006.tif]
